# Periosteum Containing Implicit Stem Cells: A Progressive Source of Inspiration for Bone Tissue Regeneration

**DOI:** 10.3390/ijms25042162

**Published:** 2024-02-10

**Authors:** Xinyuan Zhang, Chen Deng, Shengcai Qi

**Affiliations:** 1 Department of Prosthodontics, Shanghai Stomatological Hospital, School of Stomatology, Fudan University, Shanghai 200001, China; dentistzhang@yeah.net; 2 Shanghai Key Laboratory of Craniomaxillofacial Development and Diseases, Fudan University, Shanghai 200001, China; 3 State Key Laboratory of Oral Diseases, National Clinical Research Center for Oral Diseases, Department of Oral Implantology, West China Hospital of Stomatology, Sichuan University, Chengdu 610041, China; dengchendc@163.com

**Keywords:** periosteum, skeletal stem/progenitor cells, molecular regulation, bone regeneration, fracture repair

## Abstract

The periosteum is known as the thin connective tissue covering most bone surfaces. Its extrusive bone regeneration capacity was confirmed from the very first century-old studies. Recently, pluripotent stem cells in the periosteum with unique physiological properties were unveiled. Existing in dynamic contexts and regulated by complex molecular networks, periosteal stem cells emerge as having strong capabilities of proliferation and multipotential differentiation. Through continuous exploration of studies, we are now starting to acquire more insight into the great potential of the periosteum in bone formation and repair in situ or ectopically. It is undeniable that the periosteum is developing further into a more promising strategy to be harnessed in bone tissue regeneration. Here, we summarized the development and structure of the periosteum, cell markers, and the biological features of periosteal stem cells. Then, we reviewed their pivotal role in bone repair and the underlying molecular regulation. The understanding of periosteum-related cellular and molecular content will help enhance future research efforts and application transformation of the periosteum.

## 1. Introduction

The periosteum, a complex and orderly connective tissue envelope with an abundant blood supply, covers the surface of most bones. As early as 1742, Duhamel proposed the idea of periosteal osteogenesis through the observation of a bony matrix formed after implanting silver wires beneath the periosteum [1]. This was the first time that the periosteum had been described as a functional structure, thus initiating the exploration of the periosteum. Later, in the 1860s, Ollier supported this notion by discovering that, upon treatment of fractures, the integrity of the periosteum must be retained [2]. In the early 20th century, people tried to culture the periosteum in vitro and explore its osteogenic proficiency [3]. These early studies brought attention to the periosteum and, to a certain extent, uncovered the regenerative potential of the periosteum.

With ever-increasing clinical needs and constantly evolving techniques, henceforward, the periosteum has been studied extensively in the field of bone tissue repair (fracture, bone defect, etc.). Removal of the periosteum severely impairs bone healing, and successful bone graft incorporation requires the preservation of the periosteum [4]. Subsequently, the characteristic two-layer structure of the periosteum was gradually discovered [5], a structure which provides a niche for pluripotent cells and molecular factors which modulate cell behavior [6,7]. This makes it possible for the periosteum to be the indispensable contributor to callus formation in fracture repair compared to bone marrow and the endosteum [8]. Moreover, the tracing of bone development has further reinforced the innate mighty ability for bone formation of the periosteum and its cells [9]. However, due to an undetermined periosteal osteogenesis mode and the hysteresis of functional stem cell research, some in-depth mechanisms are still unclear. 

Herein, we summarize recent findings about the skeletal stem/progenitor cells of the periosteum and the molecular regulation underlying their effects. This is intended to expand our insight into the understanding of periosteal regenerative potential and then shed light into therapeutic application approaches for bone tissue repair and regeneration.

## 2. Periosteum: Its Development and Structure

The periosteum is a thin layer of tissue that covers most skeletal structures except for intra-articular surfaces and sesamoid bones. These include the bones of the cranial vault, the mandible, the maxilla, and the middle of the clavicle, formed via intramembranous ossification, as well as the skull base and the posterior part of the skull, the axial skeleton, and the appendicular skeleton, formed by means of endochondral ossification [5,10]. Bone formation begins when mesenchymal cells form condensations, within which mesenchymal cells either differentiate into osteoblasts for directly intramembranous bone formation or differentiate into chondrocytes and form an intermediate cartilage template of endochondral bone formation. The peripheral mesenchymal cells of the condensations form the perichondrium, a precursor to the periosteum. Cells from different tissues, including cartilage, the perichondrium, and the vascular endothelium, participate in this spatiotemporal event through a complicated regulation [11]. Notably, lineage analyses have shown that the perichondrium is the source of all osteoblasts, including not only cortical osteoblasts but also trabecular osteoblasts, and regulates the initiation of vascular invasion [9]. Then, the osteoblast precursors originating from the perichondrium migrate along with the invading blood vessels to form the primary ossification center during endochondral bone development [12]. 

Derived from the perichondrium, the final periosteum can be generally divided into inner and outer layers [5,13]. The inner cambium layer, contacting the bone surface, is highly cellular. It is composed of a mixed cell population potentially containing mesenchymal stem cells (MSC), differentiated osteogenic progenitor cells, osteoblasts, fibroblasts, and pericytes. The thicker outer fibrous layer’s deep section has significant elasticity since it consists of many elastic fibers. Its superficial portion contains fibroblasts dispersed in collagen fibers and rich neurovascular networks. The Sharpey’s fibers in this layer penetrate the cortex along the direction determined by tension forces from muscles and help the periosteum anchor firmly to the bone’s surface [14]. Various potential functions of the periosteum are closely related to its distinct structure.

In addition, studies reported that the two-layer structure of the periosteum is not static. It differs between bone sources in rats. The ratio of the cambium layer of the periosteum from the femur and the tibia is significantly higher than that from the rib and the calvaria [15]. Then, in rat femur, compared with the metaphyseal periosteum, the diaphyseal periosteum is much thinner, and the thickness of its cambium layer and number of cells decrease markedly with aging [16]. The human periosteum also shows site- and age-related specificity. The femoral neck surface is an exception of intra-articular surfaces where there should be no periosteum. There is significantly less cellular periosteum compared to femoral diaphysis. Between 20% and 70% of the femoral neck surface is covered by mineralized periosteum [17]. In children, the periosteum is more active, with thick periosteal layers, especially the inner cambium layer. It also contains abundant blood vessels and has a loose attachment, which explains the subperiosteal hemorrhage occurring in childhood fractures [5,14]. During natural growth, the periosteum contributes to bone elongation and modeling, and it extends alongside the appositional growth. It becomes much more closely attached to the cortex in adults. Its cambium layer is barely visible, and it contains few vessels. However, the adult periosteum still has a strong regenerative capacity and plays a vital role in bone repair such as, for example, fracture healing.

## 3. Periosteum Is a Promising Source of Skeletal Stem/Progenitor Cells

Bone is a delicate and active organ; the stem cells or early hierarchical progenitor cells it contains appear to count for a great deal in related research but also translational therapies. Periosteum has gradually aroused people’s widespread interest as a tissue with substantial skeletal stem/progenitor cells. We are attempting to determine reliable cell markers and then investigate distinct physiologic features.

### 3.1. Identification and Useful Markers

Cell markers are essential to characterize the potential stem cell nature of periosteum-derived cells (PDCs) or periosteal cells (PCs), so as to isolate the pure periosteum derived stem cell (PDSC) or periosteum derived progenitor cell (PDPC) population and the expressly functional subpopulations. 

Indeed, without exhaustive markers, PDSCs were commonly characterized by the classic MSC antigenic profile proposed by the International Society for Cellular Therapy (ISCT) in 2006: CD105-, CD73-, and CD90-positive and CD45-, CD34-, CD14- or CD11b-, CD79α- or CD19-, and HLA-DR-negative [18]. 

Further markers were then involved in the identification of PDSCs and their progenitors (Table 1). A population of self-renewing and multipotent human skeletal stem cells (hSSCs) marked by PDPN+CD146−CD73+CD164+ expression was observed to be rich in growth plate, and it also resided in periosteum [19].

With the development of immunofluorescence microscopy and lineage tracing techniques, some specific markers depicted the distinct locations of PDSCs in vivo. Furthermore, single-cell RNA-sequencing (RNA-seq) revealed more complete genetic backgrounds of PDSCs and reflected their unique signatures with relative expression status of gene markers. Subsets of periosteal Nestin+ cells and Leptin receptor (LepR)+ cells were investigated at the area of periosteum distal to the bone surface and they were abundant in 1-month and 3-month mice, respectively [20]. PCs were also characterized by paired-related homeobox gene 1 (Prx1), a marker of mesenchymal lineages in developing limbs [7]. Notably, these Prx1+ cells likely had some overlaps with Nestin+ PDCs but not LepR+ cells, which were rich in adult mice. In recent years, Cathepsin K (CtsK) labeling mature osteoclasts has been considered to characterize PDSCs and PDPCs [6,21,22]. CtsK+ PDCs included three hierarchies, CD200+CD105− PDSCs, CD200−CD105− pre-PDPCs, and CD200−CD105+ PDPCs [6,21], which were quite different from each other and other MSCs. More remarkedly, they were negative for MSC markers CD146, PDGFRα, and LepR. Single-cell RNA-seq and monocle analysis independently identified a characteristic PDSC population corresponding to that sorted by fluorescence assisted cell sorting (FACS) above. Thus, the existence of CtsK+ PDSCs was reaffirmed. In addition, Seurat analysis showed that CtsK+ PDCs clustered into four groups, varying from genotypes and functions [6,23].

**Table 1:** Markers of PDSCs/PDPCs.

		Exp.	Sites	Ref.
Minimal criteria for defining MSCs				
CD105 ^m, h, r^		+/−	tibia, femur, calvarial suture	[6,7,18,20,21,24,25,26,27,28,29,30,31]
CD73 ^h^		+	/	[18,27]
CD90 (Thy1) ^m, h, r^		+/−	tibia, femur, calvarial suture	[6,18,20,22,25,27,28,29,32]
CD45 ^m, h, r^		-	femur, phalange, tibia, calvarial suture	[6,7,18,19,20,21,24,25,26,27,28,30,31,32,33]
CD34 ^m, h^		−/low	femur, tibia	[7,18,24,27,28]
CD14 ^h^		-	tibia	[18,27,28]
CD11b ^m, h^		-	femur, tibia	[7,18,33]
CD79α ^h^		-	/	[18]
CD19 ^h^		-	/	[18,27]
HLA-DR ^h^		-	/	[18,27]
Further SC/MSC Markers				
Sca1 ^m^		+/−	femur, tibia, calvarial suture	[6,7,21,24,25,31,33]
SSEA4 ^m^		+	femur	[24]
CD29 ^m, r^		+	femur, tibia	[7,24,31,32]
MSCA1 ^h^		+	cranium	[34]
CD51 ^m, h^		+/low	tibia, femur	[6,25]
PDGFRα (CD140α) ^m, h^		+/−/low	tibia, femur	[6,7,20,25,30,31,33]
PDGFRβ (CD140β) ^m^		+	tibia, femur	[20,25,31,33]
hSSC Markers	PDPN ^h, m^, CD164 ^h, m^	+	femur, phalange	[19]
	CD146 ^h, m^	+/−/low	femur, phalange, tibia, calvarial suture, cranium	[6,19,21,26,28,30,34]
	CD73 ^h, m^	+	femur, phalange, tibia	[19,26,28]
Nestin ^m^		+	tibia, femur	[6,7,20]
LepR ^m, h^		+/−	tibia, femur	[6,7,20,30,33]
Prx1 ^m^		+	femur, tibia	[7]
Cxcl12 ^m^		+	femur, tibia	[7,30]
Gremlin1 ^m^		+	femur, tibia	[6,7,30]
CD44 ^m, r^		+	tibia, femur	[21,22,29]
STRO1 ^m, h^		+	tibia	[22,35]
CD166 ^m, h^		+	tibia	[22,26]
CD49f ^m, h^		+/low	tibia, femur	[6,21]
BP1 (6C3) ^m^, CD51 ^m, h^		−/low	tibia, femur, calvarial suture	[6]
CD200 ^m, h^		+/−	tibia, femur, calvarial suture	[6,21,30]
CD24 ^m^		+	tibia	[21]
CD106 ^h^, CD13 ^h^		+	tibia	[26]
CD271 (LNGFR, p75) ^h^		−/+	tibia, femur	[26,30]
ALP ^h^		-	tibia	[26]
Runx2 ^m^, VCAM1 ^m^		+	tibia	[30]
Endothelial/Hematopoietic Markers				
CD133 ^m^		-	femur	[24]
CD31 ^m, h^		−/low	femur, phalange, tibia, calvarial suture	[6,7,19,20,21,25,30,31,33]
Ter119 ^m^		-	tibia, femur, calvarial suture	[6,20,21,25,30,31,33]
CD235a ^h^		-	femur, phalange, tibia	[6,19,30]
CD202b (Tie2) ^h^		-	femur, phalange	[19]
MECA32 (Plvap) ^m^		+	tibia	[33]
CD3 ^m^, B220 ^m^, Gr1 ^m^		-	tibia	[33]
CD20 ^h^		-	tibia	[28]
Pericyte Markers				
NG2 ^m^		+	femur, tibia	[7]
Fibroblast Markers				
Vimentin ^m^		-	femur, tibia	[7]
D7-FIB ^h^		+	tibia	[26]
Possible Specific Marks				
CtsK ^m, h^		+	femur, tibia, calvarial suture	[6,21,22]
Sox9 ^m^		+	femur, tibia	[36,37]
αSMA ^m, h^		+	femur, tibia, calvarial suture	[30,33]
Mx1^m, h^, CCR3/5 ^m, h^		+	tibia, calvarial suture, femur	[30]
Axin2 ^m^		+	tibia	[38]

CD: cluster of differentiation; HLA: human leucocyte antigen; Sca1: stem cell antigen 1; SSEA4: stage specific embryonic antigens 4; MSCA1: mesenchymal stem cell antigen 1; PDGFRα/β: platelet derived growth factor receptor α/β; PDPN: podoplanin; STRO1: stromal cell antigen 1; LNGFR: low affinity nerve growth factor receptor; ALP: alkaline phosphatase; VCAM1: vascular cell adhesion molecule 1; Sox9: sex determining region Y-box 9; αSMA: α-smooth muscle actin. All species used, including the most frequently used mouse, are represented with corner markers. ^m^: mouse; ^h^: human; ^r^: rat. Exp.: Expression; Ref.: References.

### 3.2. Biological Characterization

Through the identification of specific cell markers, several biological features of obtained PDSC/PDPC populations were studied. Single-cell lineage analysis demonstrated that regardless of donor age, periosteal MSCs (Pe-MSCs) exhibited clonogenicity and could be expanded extensively with a fibroblast-like morphology in monolayer, maintaining linear growth curves over at least 30 population doublings. Long telomeres were detected in culture-expanded Pe-MSCs, which might contribute to their stability in culture [26]. Colony forming efficiency assay (CFE) and cell-growth analyses showed higher clonogenicity and cell growth of PDSCs compared to BMSCs, separately [7,20,32]. Under specific conditions, both parental and clonal cell populations were able to differentiate into chondrocytes, osteoblasts, and adipocytes in vitro [6,7,20,21,22,24,26,35,39]. Additionally, the multipotency of PDSCs was further highlighted by their bone and/or cartilage [6,7,20,24,26,28,40,41], as well as skeletal muscle [26] and hematopoietic marrow [28] forming capacity in vivo. Notably, recent evidence has shown that PDSCs possess the potential for self-renewal, which is a crucial feature of stem cell identity. PDSCs isolated from the initial hosts still displayed intact self-renewal and differentiation capacity, successfully undergoing subsequent rounds of transplantation [6,20].

Despite obtaining consistent conclusions, regardless of differences in selected research subjects and conditions, there are still important issues that cannot be ignored. One of these concerns is the individual differences of human donors represented by age. Even though studies have shown that in vitro, donor age was not linked to cell proliferation and differentiation [26,39], a variation in bone formation between individual donors was observed in vivo, which may include age factors [28]. In-depth studies revealed that low expression of Ki67 and high expression of p53 can serve as noteworthy markers for identifying aged human PDSCs (hPDSCs) and the expression significantly correlated with cell nitric oxide (NO) production [27]. Additionally, age affected genes involved in bone remodeling, with a significant increase in interleukin 6 (IL6) mRNA expression as well as receptor activator of nuclear factor kappa-B ligand/osteoprotegerin (RANKL/OPG) ratio [27,42]. On one hand, a larger sample size is needed to provide more reliable statistical data for comparison and identification of age-related phenotypic trends. Moreover, individual differences influence PDSC biology through more complex ways apart from age, which require further discrimination and elucidation. There was a view that the use of murine PDSCs had advantages in smaller inter-individual variability than human donors [43]. This may overcome the problem, but at the same time involves conclusion consistency issues under species diversity. In addition to the individual differences mentioned above, there are other factors that can affect the biological characteristics of PDCs, to which we need to pay attention, to have effective research and engineering strategies. Relevant experiments of rats and calves both confirmed that long bones had more osteogenic periosteum than flat bones [15,44], guiding the strategies for tissue engineering and clinical applications. While both MSCA1 and CD146 expression can be detected in cranial periosteal cells (CPCs) (Table 1), MSCA1+ cells represented a subpopulation with a higher osteogenic potential in vitro [34]. Similarly, PDGFRβ+ PDPCs showed greater colony-forming and osteogenic potential than PDGFRβ-cells, regardless of the expression of PDGFRα [25]. Researchers compared the differentiation capacity of PDSCs and BMSCs, yet their findings were conflicting. Some held a view that PDSCs had an increased osteogenic and chondrogenic potency compared to BMSCs [20]. Hayashi et al. discovered that BMSCs had a higher osteogenic capacity [32]. In a study published in Nature, it was the authors’ belief that there is no significant difference in osteogenic ability between them, PDSCs only existed as higher potential for chondrogenesis [6]. These contradictions may arise from technical differences between the studies, with cell sorting via various markers being a crucial factor. Therefore, this needs to be well considered for selecting valid markers. 

In terms of the direction of differentiation, it is noteworthy that Stro1+ PDPCs could be cultured for up to 10 passages without loss in population numbers or the ability to form mineralized tissue. However, late passage cultures were osteogenic and lost chondrogenic markers [18]. It demonstrates the low original chondrogenicity of cultured PDPCs. To maintain consistent chondrogenic capacity, specialized media, such as combining micromass culture and transforming growth factor β (TGFβ) 1 treatment, are required for culture cells [45]. A single-cell RNA-seq dataset generated recently showed that chondrocyte populations had low expression of fatty acid oxidation (FAO) genes compared with osteoblasts. As a result, the use of lipid-reduced serum (LRS) promoted chondrogenic differentiation of PCs in micromass or pellet cultures [46]. While osteogenic differentiation can occur without specific culture, it was proved that the addition of dexamethasone (Dex) and fetal bovine serum (FBS) to the culture media was crucial for alkaline phosphatase (ALP) expression of human PDCs (hPDCs) during early differentiation stages. Then, for the expression of the main transcription factors governing osteogenesis and following differentiation towards mature osteoblasts, the subsequent combination of trans-retinoic acid (atRA), FBS, Dex, and BMP2 was required [47]. It is evident that the culture additives to isolated PDCs have profound impacts on cell differentiation and need to be carefully controlled. Inorganic ions also have a potential biphasic effect, where an optimal level of released calcium positively affects hPDSCs, but excessive concentrations have a negative effect on terminal osteo-chondrogenic differentiation [41]. This finding provides guidance for the use of scaffolds with the proper phase of ions in bone engineering. An oxygenated environment is another essential factor for hPDC survival and maintenance of osteogenic differentiation potency [48]. In vitro culturing under hypoxia (0.1% oxygen) likely did not affect cell proliferation but significantly enhanced PDPC chondrogenic differentiation while reducing osteogenic differentiation [49]. Last but not least, PDPCs have an osteogenic response to mechanical stimulation [45], highlighting the sensitivity of periosteum to mechanical signals and the potential for applications in distraction osteogenesis.

Indeed, there are intricate mechanisms together with complicated interaction networks through which these biological characteristics are exerted and then show great contributions to bone formation or regeneration.

## 4. The Dominant Role of Periosteum in Fracture Healing

The process of normal bone healing after a fracture recapitulates the well-defined stages of bone formation during embryogenesis, except for inflammatory response and mechanical loading. Specifically, it starts with hematoma formation, which triggers an inflammatory response [42]. Following the production and release of several important molecules contributing to mesenchymal stem cell migration and proliferation, this is followed by differentiation into chondrocytes and osteoblasts in order to generate a primary callus. The soft callus later undergoes revascularization and calcification, and it is finally remodeled to fully restore a normal bone structure without the formation of scar tissue [50].

Diverse tissues together with the cells they contain have been proposed to participate in the process of bone repair after a fracture and to play integral roles. Bone marrow, for example, provides niches for both hematopoietic and mesenchymal stem cells. Through the approach of transplantation, they are proved not to be main contributors to osteogenesis and chondrogenesis, but to have specific effects on inflammatory and matrix remodeling [51]. Another study identified that BMSCs also possessed angiogenic differentiation potency and induced the formation of large vessels (diameter > 50 μm) during fracture [52]. Endothelial cells, which are the predominant lineage of cells involved in angiogenesis, do not seem to transdifferentiate into skeletal progenitors during fracture healing, as cartilage and bone within the callus are not derived from Tie2-expressing cells. However, it is important to note that angiogenesis is crucial for normal fracture healing, and it is closely related to osteogenesis [46,52]. In addition, other sources of cells such as stem cells from muscle [53] and adipose tissue, as well as pericytes from the surrounding blood vessels, are involved in bone repair as well [54]. 

With constant in-depth study, it was ultimately established that progenitor cells from the periosteum are the primary contributors to the callus during fracture healing [4,37]. Initially, PDSCs/PDPCs are recruited to the injury site through specific mechanisms. Mx1+/αSMA+ periosteal skeletal stem cells (PSSCs) were identified specifically to express CCL5 receptors, CCR3 and CCR5, and induce PSSC migration [30]. Additionally, macrophages play an important role in the process through interaction with periosteal components. On one hand, M1 (pro-inflammatory) phenotype macrophages are induced by the inflammatory cytokines released during acute inflammation. They have phagocytic and clearance properties but have disadvantages of inducing chronic inflammation and of delaying healing [55]. Thus, the transition from pro-inflammatory M1 to M2 (anti-inflammatory) phenotypes at the early stages of bone injury is a prerequisite to successful bone healing. Periosteal extracellular matrix (PEM) was identified as a participant in macrophage chemotaxis and the M2 polarization process [56]. On the other hand, periosteal tartrate resistant acid phosphatase (TRAP) positive mononuclear cells were observed to recruit Nestin+ and LepR+ PDCs to the surface of cortical bone [20]. Colnot [8] performed cell lineage analyses after transplanting bone grafts and demonstrated that even when the tissues were transplanted ectopically, the majority of cells were locally recruited for bone repair. It was subsequently confirmed that PCs and BMSCs in skeleton formation were also locally derived [7]. These results indicate that bone itself is the main source of cells for bone formation and repair.

After recruitment, periosteal progenitors undergo proliferation and differentiate into osteoblasts and chondrocytes via various molecular signals. As with the development, bone healing relies on two robust ossification processes, intramembranous ossification and endochondral ossification. Lineage analyses showed that periosteum, bone marrow, and endosteum all gave rise to osteoblasts. Chondrocytes within fracture callus, however, were primarily derived from periosteum, indicating that periosteum supports both chondrogenesis and osteogenesis, whereas bone marrow/endosteum only supports osteogenesis during bone repair. These explain to a certain extent the program of bone healing observed after periosteal or bone marrow/endosteal injuries, which are healed by endochondral and intramembranous ossification, respectively [8]. Indeed, during normal bone homeostasis, PDSCs generate osteoblasts and specialize in intramembranous bone formation [6]. Unfortunately, little is known about how injury stimulates PDSCs to generate chondrocytes and to contribute to the endochondral process of fracture, nor is it understood whether a specific periosteal progenitor population drives the formation of the cartilage callus or the signaling pathways involved.

Particularly, some fractures can heal in the absence of a cartilage callus, and this may depend on environmental stability. For instance, in cases where the fracture is not stabilized, the formation of a large cartilage callus appears to be required in large-scale bone regeneration, whereas fractures that are rigidly stabilized heal without any apparent cartilage formation [57]. In addition, the correlation between fracture healing and bone development is also reflected on this issue. When mandibular and tibial PDPCs were transplanted to mandibular defects, intramembranous ossification and endochondral ossification were observed, respectively [58]. Moreover, when the periosteum was collected from the cranium and the radius to create subcutaneous bone, the direction of osteogenesis differed between the two groups [44]. The embryonic origins of them are different, which perhaps determines progenitor cell fate, and ossification probably occurs under different genetic controls [58]. Recently, a study transplanted autologous bone grafts into femoral defects in mice. During the healing, PDPCs near the host-graft border formed cartilage, while cells in the center differentiated directly into osteoblasts [46]. Periosteal chondrogenesis can be further interpreted as a secure healing method with the lack of exogenous lipids, linked to poor vascularization. Another aspect affected by vascularization is the oxygen content in the environment. The ability of PDPCs to differentiate into chondrocytes was significantly improved under hypoxic conditions. This explains why the regions close to the fracture gap within the thickened periosteum undergo endochondral ossification, while the distal regions heal through intramembranous ossification [49,59]. 

There are still a lot of uncertainties about these two bone healing pathways. Aided by Cre-based lineage trace, cells marked by expression of CtsK [60], periostin [7], αSMA [30,33], Axin2 [38], and Sox9 [36,37] were found in periosteum. Once again, they proved that PDSCs/PDPCs mainly contribute to fracture callus formation. However, sometimes they do not behave exactly the same through specific molecular regulation.

## 5. Molecular Signaling Pathways Regulating Periosteal Effects

It has become gradually clear that the periosteum plays a central role in bone development and regeneration. Researchers are actively investigating the intricate molecular regulation and signaling interactions involved in these processes. With the development of new approaches, studies have revealed the effects of several signaling pathways, such as BMP, PDGF, Ihh, Wnt, Notch signaling, etc. This knowledge is particularly valuable as it provides insight into effective treatment strategies for enhancing bone repair and reconstruction.

### 5.1. BMP Signaling

Bone morphogenetic protein (BMP) signaling functions rely on TGFβ superfamily ligands, type I and type II BMP receptors, and canonical drosophila mothers against decapentaplegic protein (Smad) downstream pathway [61]. It has been extensively investigated in the field of bone biology. Although several BMPs are expressed spatiotemporally in bone and act as essential motivators in bone developmental and regenerative processes, BMP2 has a unique role in PDPCs, and thereby regulates BMP signaling inducing periosteal bone formation, growth, and fracture repair. Neither absence of other BMPs such as BMP4, BMP7, and BMP9 [62] nor BMP2 loss in mature osteoblasts causes spontaneous fractures [63]. Yet, both BMP2 and BMP4 expressions are increased and activate osteogenesis when PDCs respond to mechanical stretch, along with reduction in BMP6 expression [64]. BMP6 is related to the expression of IL1β and IL6, which are involved in RANKL-dependent osteoclastogenesis and bone resorption [42]. This might represent a special profile of mechanical stress-driven bone remodeling.

Additionally, strong increases in BMP2 expression and activated signaling were observed at an early stage of bone forming induced by implanted hPDCs [41]. Interestingly, when human recombinant BMP2 (hrBMP2) was loaded, endochondral bone formation was observed but not original intramembranous ossification [40]. These phenotypes suggest that BMP2 signaling is involved in both forms of periosteal ectopic bone formation.

**Figure 1:**
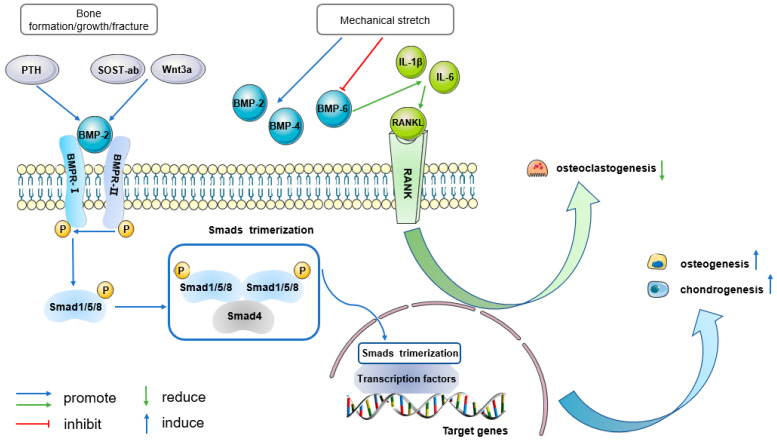
BMP signaling represents a fundamental signaling profile in periosteal effects. BMP2 signaling is proposed to function downstream of PTH and the canonical Wnt signaling pathway during bone growth and fracture repair. During bone formation and fracture healing, BMP2 signaling specifically acts on PDPCs (blue pathway). After ligand binding, phosphorylated (P) BMPR-II helps to phosphorylate BMPR-I. Activated BMPR-I recruits and phosphorylates pathway-specific receptor activated Smads (Smad1/5/8), which can form trimers with Smad4 and translocate into the nucleus. Inside the nucleus, the Smad trimerization recruits and/or integrates with cell type specific transcription factors and then regulates target gene expression. Thereby, BMP2 signaling increases periosteal osteogenesis and chondrogenesis. When PDCs respond to mechanical stretch, the expressions of BMP2 and BMP4 increase, while BMP6 expression is suppressed. BMP-6, in turn, induces the expressions of IL1β and IL6, which promote osteoclast differentiation through the RANKL-dependent pathway (green pathway). In this way, PDCs sense mechanical stress and promote osteogenesis but decrease osteoclastogenesis. PTH: parathyroid hormone; SOST-ab: sclerostin neutralizing antibody; BMP: bone morphogenetic protein; BMPR-I: type I BMP receptor; BMPR-II: type II BMP receptor; RANKL: receptor activator of nuclear factor κ-B ligand; RANK: receptor activator of nuclear factor κ-B; P: phosphorylation.

BMP2 signaling is also essential for the initiation of fracture healing [62,65]. A transgenic BMP2-deficient mouse model showed that local expression of BMP2 played a critical role in osteogenic and chondrogenic differentiation of PDPCs during repair, which eventually induced robust periosteal bone/cartilage callus formation [62,66]. The same conclusion was verified in bone autograft healing [67] (Figure 1).

BMP2 signaling provides a founding signaling profile in periosteal effects, but further studies are needed to elucidate the mechanisms and signal interplay involved. Research suggested that BMP2 signaling may act downstream of parathyroid hormone (PTH) and canonical Wnt signaling pathway activation during periosteal growth and fracture repair [63]. Meanwhile, the synergistic interaction of BMP2 signaling with other pathways also holds significant importance and offers valuable insights for clinical treatment.

### 5.2. PDGF Signaling

Platelet-derived growth factor (PDGF), which is released by platelets and macrophages at fracture sites during initial inflammation, has potent mitogenic and chemotactic effects and enhances angiogenesis. The effects of PDGF are mediated through the specific receptor tyrosine kinases (RTKs) PDGFRα and PDGFRβ, which form both homodimers and heterodimers [68]. An increasing number of PDPCs express PDGFRβ during fracture repair, while very few PDGFRα+ cells are in the periosteum or fracture callus, and most of them co-express PDGFRβ. This expression status is sustained both in vitro and in vivo, suggesting that PDGFRβ is the main pathway for PDGF signaling in periosteum [25,31].

**Figure 2:**
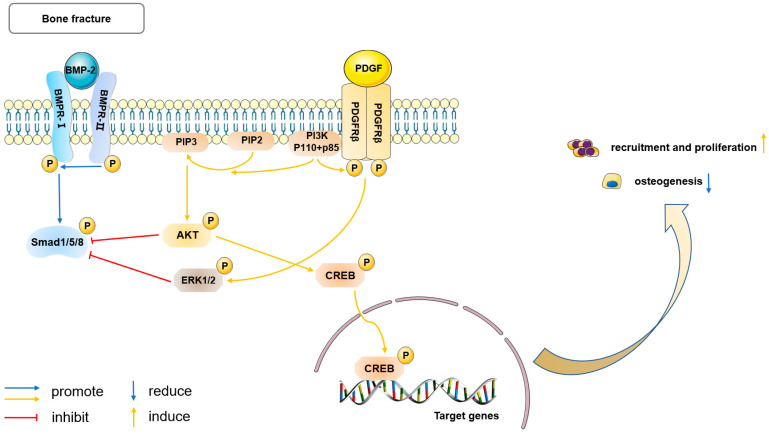
PDGF signaling mediates periosteal activation and blocks BMP2-induced osteogenesis. PDGFRβ, a kind of receptor tyrosine kinase (RTK), plays a crucial role in the PDGF signaling in the periosteum. There is mutual interaction between PDGF/PDGFRβ and PI3K/AKT signaling (yellow pathway), which is essential for activating PDCs following fracture. On the one hand, after PGDF combines to RTK (PDGFRβ), PI3K directly binds to phosphorylated (P) PDGFRβ via the regulatory domain (p85), and is therefore targeted to the inner cell membrane. Binding of p85 subunit of PI3K to the phosphorylated RTK leads to conformational changes in the catalytic domain of PI3K (p110) and consequently promotes the conversion of PIP2 to PIP3. PIP3 contributes to AKT phosphorylation and activation. The transcription factor CREB is directly phosphorylated by activated AKT, p-CREB then transports into the nucleus and regulates target gene transcription. On the other hand, upregulation of PI3K sensitizes PDCs to the effects of PDGF and enhances phosphorylation of PDGFRβ. Through this synergy, the PDGFRβ-PI3K signaling axis exerts its anti-apoptosis, pro-cell proliferation and recruitment effects. The binding of PDGF to its receptor, PDGFRβ, stimulates both the PI3K/AKT and ERK1/2 pathways in PDCs. Notably, it blocks the canonical BMP-2/Smad pathway (blue pathway), inhibiting periosteal osteogenesis. PDGF: platelet derived growth factor; PDGFRβ: PDGF receptor β; PI3K: phosphoinositol-3-kinase; PIP2: phosphatidylinositol-4,5-bisphosphate; PIP3: phosphatidylinositol-3,4,5-trisphosphate; CREB: cyclic adenosine monophosphate (cAMP) response element-binding protein. ERK1/2: extracellular signal-regulated kinases 1/2; P: phosphorylation.

Trap-Cre PDGF-BBfl/fl mice and conditional knockout of PDGFRβ in PDCs demonstrated that macrophage-lineage TRAP+ mononuclear cells secreted PDGF-BB to recruit Nestin+ and LepR+ PDCs, predominantly located in the outer layer to the periosteal surface for cortical bone formation and regeneration in the bone defect region [20]. PDGF strongly increases proliferation and decreases apoptosis of PDPCs in vitro [25]; in vivo, the effects are similar, as PDGF/PDGFRβ mediates periosteal activation and progenitor proliferation during the early phases of fracture healing through an intact interaction with the PI3K/AKT signaling pathway [31]. The direct effects of PDGF on osteogenesis, however, remain controversial and depend on the cell type [69]. In vitro studies showed inhibitory effects of PDGF on BMP2-induced osteogenic differentiation in PDPCs through activating ERK1/2 and PI3K/AKT signaling to reduce the canonical Smad phosphorylation and BMP target gene expression (*Dlx5* and *Id1*) [25]. However, due to the stepwise nature of bone repair and the complex physiological environment in vivo, the cross-talk between PDGF and BMP2 signaling may not be so absolute or simplex. They may play distinct roles at different stages of fracture healing. Specifically, cell recruitment and proliferation in the earlier stage, promoted by PDGF, could be essential for the following osteogenic differentiation induced by BMP2, so as to achieve a delicate balance and then the most effective fracture healing [70]. This hypothesis was verified in BMSCs [71] and should be reasonably extrapolated to PDSCs, but more work is needed to fully confirm and elucidate it (Figure 2).

Therefore, PDGF signaling has shown a variety of potential functions in periosteum-mediated fracture healing. Its interaction with other signaling pathways, such as BMP2 signaling, will provide it with broader applications.

### 5.3. Ihh Signaling

Indian hedgehog (Ihh) is one of the three mammalian homologues of Hedgehog (Hh) protein, and the other two members are Desert Hh (Dhh) and Sonic Hh (Shh). Ihh signaling plays a critical role in the development and repair of bones. It also interacts and cooperates with other molecules in a complex manner. Ihh signaling is indispensable for the differentiation of osteoblast in perichondrium during the endochondral bone development in embryos [72]. In the absence of Ihh signaling, the progenitor cells residing within the perichondrium tend to undergo chondrocyte differentiation and fail to contribute to osteoblasts in either the bone collar or the primary spongiosa. In adult, Ihh/Shh signaling targets mesenchymal lineages in early periosteal callus and is required for periosteal bone repair [24]. Interestingly, these two processes are speculated to be related to the synergistic effect between Hh and BMP signaling through in vitro experiments. Remarkedly, this synergy has been shown to be associated with osteogenic differentiation, as well as with both osteogenic and chondrogenic differentiation [24,73].

**Figure 3:**
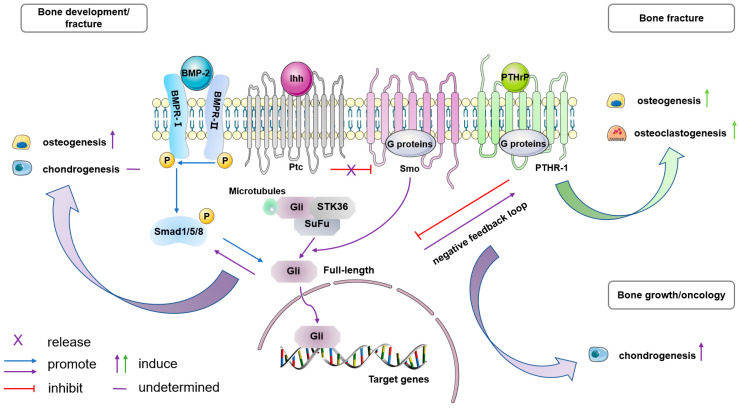
Ihh signaling plays a critical role in periosteal bone biology and shows complex interaction with other molecules. Ihh is one of the three mammalian homologues of Hh protein (violet pathway). Ptc, a twelve-pass membrane protein, binds the Ihh ligand. In the absence of the ligand, Ptc interacts with and inhibits Smo, a seven-pass membrane G-protein coupled receptor (GPCR). When Ptc binds Ihh, its inhibitory effect on Smo is released, activating Smo. Then, Smo promotes the disbandment of the Gli-composite complex from microtubules, allowing full-length Gli to enter the nucleus and act as a transcriptional activator. During bone development and fracture repair, it is presumed that there is a synergy between Hh and BMP signaling (blue pathway). Together they enhance osteogenesis. However, in terms of chondrogenetic effect, the conclusion has not yet been determined. Ihh signaling also has several key functional interactions with PTHrP (green pathway). In fracture healing, PTHrP binds its receptor (PTHR1), a GPCR, and mediates periosteal osteoblastic and osteoclastic activity. During the growth of long bones, Ihh induces PTHrP expression. In turn, PTHrP delays the production of Ihh. Ihh/PTHrP, acting together, initiate a negative feedback loop that sustains chondrogenesis and generates a cartilage tumor. Hh: Hedgehog; Ihh: Indian hedgehog; Ptc: Patched; Smo: Smoothened; SUFU: suppressor of Fused; PTHrP: parathyroid hormone (PTH)-related peptide; PTHR1: type I PTH receptor; P: phosphorylation.

Ihh signaling has several key functional interactions with PTH-related peptide (PTHrP). PTHrP resides in the fibrous layer of periosteum and its receptor (PTHR1) is in the subjacent cambial layer [74]. Together, Ihh and PTHrP control the rate at which chondrocytes proliferate and differentiate, thereby driving linear bone growth through a feedback loop [11]. Ptpn11 deletion in Ctsk+ PDPCs upregulated Ihh/PTHrP signaling, leading to excessive proliferation and chondrocyte differentiation, finally causing chondroid neoplasm metachondromatosis [22]. Impaired healing of tibial fracture in the PTHrP cKO mouse results from the failure of PTHrP induced osteoblastic activity and osteoclastic remodeling on the periosteal surface, and can be further explained by abnormal interactions among PTHrP, Ihh, and BMP signaling pathways [74,75] (Figure 3). 

*Sox9* is considered a key regulator gene in the process of chondrogenesis. Previous research showed that *Sox9*+ cells present in the periosteum of long bones possess the capability to differentiate into chondrocytes and osteoblasts, contributing to the formation of callus after bone fractures [36,37]. Recent studies also confirmed the presence of *Sox9*+ periosteal cells in the rib, which act as messenger cells that require Ihh/Shh signaling for activation. These cells then stimulate neighboring cells to differentiate into a hybrid osteo-chondral callus [76]. Although extensive research has been conducted on the involvement of Ihh signaling in various physiological processes related to the periosteum [77], conflicting results have prevented a clear determination of its role in directing the differentiation of PDPCs, for instance.

### 5.4. Biological Characterization

Wnt signaling was shown to function downstream of Ihh signaling in endochondral bone development during mouse embryogenesis [78]. The Wnt ligand Wnt7b expresses specifically in perichondrium dependent on Ihh signaling and promotes osteoblast differentiation through the canonical β-catenin pathway. Remarkedly, Wnt7b is capable of inducing vascularization of the hypertrophic cartilage in *Ihh^−/−^* mice [79].

**Figure 4:**
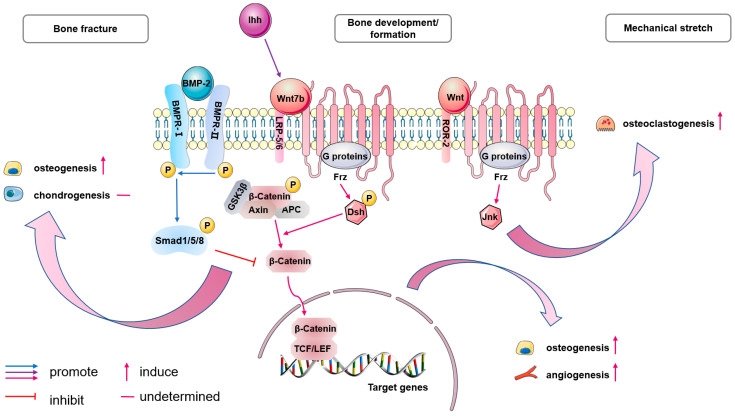
Wnt signaling has important indirect as well as direct effects on PDCs. Wnt signaling is involved in endochondral bone development (pink pathway). One of its ligands, Wnt7b, is expressed in the perichondrium dependent on Ihh signaling (violet pathway). The Wnt ligand signals via a seven transmembrane G-protein coupled receptor (GPCR), Frz, together with LRP5/6 co-receptors, initiating canonical Wnt signaling pathway. The Frz receptor leads to phosphorylation and activation of the Dsh protein. The activated Dsh inhibits the combination ability of GSK3β, leading to accumulation of free and unphosphorylated β-Catenin in the cytoplasm, which then translocates to the nucleus. There, β-Catenin binds to TCF/LEF, promoting changes in the transcriptional machinery that leads to expression of several target genes. Eventually, Wnt signaling promotes osteogenesis and angiogenesis during bone development and formation. Interestingly, in the injured periosteum, BMP2 (blue pathway) represses β-catenin-dependent Wnt signaling to induce chondrogenic cell fate determination. In part, it indicates the pro-osteogenesis and anti-chondrogenesis capacity of Wnt signaling. However, Wnt signaling is also demonstrated to act on PDCs and induce both osteogenesis and chondrogenesis. Thus, Wnt signaling enhances osteogenesis, its effect on chondrogenesis is not clear. β-catenin-independent pathway (non-canonical pathway, pink pathway) is activated when PDCs are loaded with mechanical stress. The binding of Wnt ligands to Frz receptor and co-receptor ROR2 induces osteoclastogenesis through Jnk. Frz: Frizzled; LRP5/6: lipoprotein receptor-related protein 5/6; Dsh: Disheveled; TCF: T-cell factor; LEF: lymphoid-enhancing factor; Jnk: c-jun N-terminal kinase; P: phosphorylation.

Wnt has been identified as the key factor in the early phase of the bone formation induced by transplanted hPDCs. Different phosphorylated and active β-catenin expression affects the final bone forming effect [41]. Additionally, the periosteum is known to sense mechanical stress and promote osteogenesis. Stretch-stimulated progenitors enhance osteoblast differentiation through regulating the expression levels of several Wnt-related genes. During the healing of tibial fracture in mice, Wnt-responsive cells (WRCs) were reported to be located on the endosteal surface of bone contributing to intramembranous bone repair [80]. Whereas in the injured periosteum, rBMP2 represses β-catenin-dependent Wnt signaling, resulting in Sox9 upregulation; consequently, cells in the injured periosteum adopt a chondrogenic fate, and thus, repair heals via endochondral ossification. This indicates that Wnt and BMP2 signaling may have bone compartment-specific effects and diversely promote bone healing. Notably, another study later traced WRCs expressing Axin2, a negative regulator of Wnt signaling, on the periosteum of the tibia in mice [38]. This subset of PDPCs is activated upon injury and gives rise to both cartilage and bone (Figure 4).

Thus, the underlying role of Wnt signaling in other molecular pathways partly explains its importance in periosteum or perichondrium. Meanwhile, its direct effects are being gradually understood, so its application strategies for periosteal bone formation and repair may be more effective in the future.

### 5.5. Notch Signaling

The role of Notch signaling in different cell lineages has been extensively studied, revealing complex results. Endothelial-cell-specific Notch signaling promotes angiogenesis and couples it to enhanced osteogenesis in bone. In osteo-lineage, a negative effect of the Notch ligand, Jagged1 (Jag1) was observed specifically on the differentiation stage of mesenchymal progenitor cells toward osteoblasts [81,82]. Additionally, the effects of Notch signaling trough Jag1 appear to be compartment-dependent. It represses periosteal osteogenesis of cortical bone [81]; its regulation in trabecular bone has not been determined [81,82].

The role of Notch signaling in PDPCs remains controversial. Notch signaling inhibits differentiation of αSMA-labeled PDPCs into osteogenic lineages in vitro, indicated by a lack of expression of bone sialoprotein and osteocalcin, and accompanied with culture overgrowth. In vivo, implanted PDPCs fail to form ectopic bone healing [33]. In a recent study, during the process of osteogenic tumor formation caused by *Lkb1* deletion in *CtsK*+ PDPCs, Notch target gene *Hey1* upregulated together with oncogene *Mdm2*, suggesting that Notch signaling may be involved in excessive osteogenesis [21]. However, bulk RNA-seq analysis of *Ctsk*+ PDCs showed low level expression of Notch related genes [6]. 

Taking all these together, it can be reasonably inferred that the specific function of Notch signaling is likely to be contextual and relates to distinct cell lineages or subsets. Whether there is strong target regulation by Notch signaling towards PDCs needs further studies to elucidate.

### 5.6. Other Related Molecular Regulation

In addition to the above signaling pathways, periosteal effects are also mediated by some other molecules. Although further studies are needed, they may provide some insight into a new forefront in periosteal bone-regenerative understanding and therapeutic strategies.

Periostin resides adjacent to the periosteal bone surface with type H vessels during cortical bone growth and diminishes during late adulthood [20]. Periostin is indispensable for maintaining the pool of PCs and contributes to efficient periosteal bone repair [7]. Furthermore, PDGF-BB induces periostin expression exactly to generate a periosteal osteogenic microenvironment for differentiation and adhesion of PDCs [20]. These fundings highlight the precise regulation of periostin in PDCs, which warrants further investigation and potential applications in clinical settings. 

As angiogenesis and osteogenesis are closely interconnected, vascular endothelial growth factor (VEGF) plays a critical role in bone repair. Interestingly, transgenic deletion of VEGFA in osteoblast lineage cells, but not in endothelial lineage cells, led to both impaired intramembranous ossification and angiogenesis during tibia cortical defect repair [83]. Moreover, VEGFA from early osteo-lineage cells (Osterix+), rather than mature osteoblasts, is critical for periosteal angiogenesis and woven bone formation during fracture repair [84]. In addition, the expression of four VEGF isoforms (VEGF121, VEGF165, VEGF189, and VEGF206) and two receptors (VEGFR1 and VEGFR2) was observed in cultured hPDCs. VEGF stimulates the osteoblastic differentiation of hPDCs with elevated ALP activity and increased Runx2 transactivation [85]. Thus, it is truly necessary to clarify the role of VEGF in PDCs during periosteal fracture healing in vivo.

ε-Aminocaproic (EACA) acid, an inhibitor of plasminogen, promotes periosteal osteogenesis and inhibits periosteal chondrogenesis during appendicular bone fracture healing, leading to a biomechanically stronger callus. TGFβ, BMP, and Wnt signaling was proved to be involved in this process [86]. It reveals that hematoma stabilization and enhanced periosteal bone healing can be implemented simultaneously, offering a promising new clinical therapy.

The periosteum, a connective tissue covering bone surfaces, contains sensory nerves that release neuropeptides. One of these, calcitonin gene-related polypeptide (CGRP), was shown to promote osteogenic differentiation of PDSCs in rats through CGRP-receptor-coupled cyclic adenosine monophosphate (cAMP) activation of binding protein 1 (CREB1) and SP7 (also known as Osterix), and eventually improved bone fracture healing [29]. These findings provide insights into the local neuronal regulation of periosteum.

As suggested above, multiple signaling pathways and molecules work in tandem to create a complex network that influences the behavior of pluripotent cells within the periosteum. Certainly, although it still contains combined spatial and temporal regulation, the deeper mechanisms need further elucidation.

## 6. Discussion

In this review, we explored retrospectively the developmental origin of the periosteum and illustrated its unique structure. Through a reasonably deep understanding of the structure, we believe that PDSCs/PDPCs in the cambium layer play crucial roles in various functional activities, especially in bone regeneration. We further reviewed their markers, cell behavior, and molecular modulation. Our findings provide conceptual insights, but further discussion is needed to explore useful markers and signatures associated with these cells. 

The periosteum is believed to be a reservoir for skeletal stem cells (SSCs), although previous research on SSCs primarily focused on characterizing BMSCs. As illustrated, they vary from PDSCs in several aspects. In terms of differentiation potential, when BMSCs and PDSCs were selected for CD29, CD90 and against CD45, the former had slightly higher osteogenic ability than the latter [32]. PDSCs gradually display advantages when more specific cell markers are used to isolate the PDSC population that may be more purified. Another study used Prx1 and showed that similar osteogenic potential between the two sources of pluripotent cells and PDSCs had an increased chondrogenetic potential compared to BMSCs [7]. PDSCs even showed higher potency both osteogenic and chondrogenic when using Nestin and LepR for cell sorting [20]. Also, their roles in fracture healing are not the same [8]. Although it is undeniable that BMSCs show essential effects [52,87], the major contributor to callus formation was identified as PDSCs and periosteum preservation is crucial for successful bone repair [4,37]. Despite some inconsistencies, PDSCs and BMSCs show excellent ability to promote regeneration in comparison with other MSCs, for example, adipose tissue-derived MSCs (AMSCs) [32]. Taking all these together, we hypothesized that PDCs and BMSCs from their distinct habitats, may have specific differentiation mechanisms and innately different contribution to bone repair. Considering they are both clinically useful SSCs, to maximize cross-study comparison but to minimize variability of research methods like marker selection is eagerly anticipated, so that we can truly concentrate on the differences in cell fate decisions between PDSCs and BMSCs and boost more effective and targeted application strategies.

Based on the grasp of periosteal osteogenesis and the inseparable relationship between the skeletal and immune system, it is reasonable to presume that periosteum is involved in osteoimmunology. This is verified by some facts. The immune regulation of osteoclasts accounts for an important part of osteoimmunology, and RANKL is a main conjunction factor [88]. The age-related changes of PDSCs [27] and the mechanisms of periosteal mechanical response [89] appear to be partly regulated by RANKL-mediated osteoclast activity and bone remodeling. Macrophages, another linker from the immune system, have shown a vital role in periosteum-dominated fracture repair. Transition from classically activated M1 macrophages to alternatively activated M2 macrophages is promoted by PEM which determines the beginning of successful fracture healing [55,56]. Subsequently, TRAP+ macrophage cells are involved in the recruitment of PDCs to the periosteal surface, where the recruited PDCs then undergo osteoblast differentiation coupled with vascularization [20]. The scope of osteoimmunology has encompassed a wide range of interactions, not only including those between osteoclasts and immune components, but also osteoblasts and osteoclasts, osteoblasts and hematopoietic cells, while more communication factors were also identified [42]. The specific role and underlying mechanisms of periosteum and its cells in this field need further elaboration.

Since periosteum shows an outstanding repairing effect and regenerative ability, with the rise and development of tissue engineering techniques, people have been trying to find substitutes mimicking periosteum. At the beginning, many different combinations of biological and polymeric materials were investigated for macrostructural bionics of periosteum including acellular human dermis [90], cell composite hydrogel periosteum [91], and cell sheets [92]. Along with the thorough study and continuous advance of techniques, the imitation of periosteum has become more microscopic. Precise topographic cues (microgroove patterns) from native periosteum can be replicated by micropatterning techniques, which helps cell alignment of the stem cells laden onto them and simulates natural periosteum for bone repair therapies when they are adhered onto various scaffolds [93,94]. With the help of another key technology, electrospinning, a variety of biomaterials were used in different manners for diverse functional artificial periosteum manufacture [95,96,97]. Notably, the structural development of periosteum was maximally simulated, and a kind of bionic periosteum was able to make periosteal reconstruction and bone repair become more natural in recent times [98]. The investigation of periosteum substitutes is accelerating towards the perspective of structural and functional regeneration of periosteum, as well as towards an innovative impact on the design and translation of bone tissue engineering. 

In conclusion, we underline the potential of periosteum and its pluripotent stem cells as promising inspiration in bone biology. In light of in-depth, ongoing studies on periosteum, we firmly believe that it will be widely used in bone regeneration and repair by virtue of its unique advantages.

## Data Availability

This is a review article. Data from the research papers that are cited are available on request.
